# The Role of Inflammation in Breast and Prostate Cancer Metastasis to Bone

**DOI:** 10.3390/ijms22105078

**Published:** 2021-05-11

**Authors:** Andy Göbel, Stefania Dell’Endice, Nikolai Jaschke, Sophie Pählig, Amna Shahid, Lorenz C. Hofbauer, Tilman D. Rachner

**Affiliations:** 1 Mildred Scheel Early Career Center, Division of Endocrinology, Diabetes, and Bone Diseases, Department of Medicine III, Technische Universität Dresden, 01159 Dresden, Germany; Teresa.DellEndice@uniklinikum-dresden.de (S.D.); Nikolai.Jaschke@ukdd.de (N.J.); Sophie.Paehlig@ukdd.de (S.P.); Amna.Shahid@ukdd.de (A.S.); Lorenz.Hofbauer@ukdd.de (L.C.H.); Tilman.Rachner@ukdd.de (T.D.R.); 2 German Cancer Consortium (DKTK), Partner Site Dresden and German Cancer Research Center (DKFZ), 69120 Heidelberg, Germany; 3 Center for Healthy Aging, Technische Universität Dresden, 01159 Dresden, Germany

**Keywords:** inflammation, metastasis, cancer, bone colonization, tumor microenvironment

## Abstract

Tumor metastasis to bone is a common event in multiple forms of malignancy. Inflammation holds essential functions in homeostasis as a defense mechanism against infections and is a strategy to repair injured tissue and to adapt to stress conditions. However, exaggerated and/or persistent (chronic) inflammation may eventually become maladaptive and evoke diseases such as autoimmunity, diabetes, inflammatory tissue damage, fibrosis, and cancer. In fact, inflammation is now considered a hallmark of malignancy with prognostic relevance. Emerging studies have revealed a central involvement of inflammation in several steps of the metastatic cascade of bone-homing tumor cells through supporting their survival, migration, invasion, and growth. The mechanisms by which inflammation favors these steps involve activation of epithelial-to-mesenchymal transition (EMT), chemokine-mediated homing of tumor cells, local activation of osteoclastogenesis, and a positive feedback amplification of the protumorigenic inflammation loop between tumor and resident cells. In this review, we summarize established and evolving concepts of inflammation-driven tumorigenesis, with a special focus on bone metastasis.

## 1. Introduction

### 1.1. The Good and the Bad of Inflammation

Inflammation is an evolutionarily conserved mechanism to protect organisms from invading pathogens, to clear damaged cells, and to repair injured tissues, ultimately aiding in restoring homeostasis [1]. A prime example of inflammation-evoking stimuli are bacterial infections, where an innate immune response is triggered by the recognition of specific exogenous signals, referred to as pathogen-associated molecular patterns (PAMP) by pattern-recognition receptors, which are expressed on myeloid and endothelial cells, fibroblasts, and lymphocytes [2]. The resulting reaction is mediated by soluble factors (i.e., cytokines and chemokines) and cellular components, culminating in a variety of effects. These comprise recruitment, extravasation, and activation of several immune cells including neutrophils and macrophages, phagocytosis of bacteria, as well as the production of inflammatory, chemotactic, cytotoxic and tissue-degrading mediators [1,2,3]. Acute inflammatory reactions need to be precisely controlled to prevent excessive, unwanted host tissue damage. Therefore, engagement of inflammatory pathways is typically followed by a resolution and tissue repair phase [1,3]. This is ensured by the self-limiting activation of regulatory cells and anti-inflammatory cytokines and by a switch of lipid mediators, where lipoxins, resolvins, and protectins serve as stop signals. These molecules limit further recruitment of immune cells and vascular permeability, promote infiltration of wound-healing monocytes, and induce clearance of dead neutrophils [3,4,5]. Unresolved acute inflammation can progress in chronic, pathological inflammation with the manifestation of diseases, continuous destruction of host tissue, and the disturbance of whole body homeostasis [4]. Chronic inflammation can be subdivided into high-grade and low-grade forms leading to multifaceted clinical consequences including rheumatoid arthritis, inflammatory bowel disease, asthma, psoriasis, metabolic syndrome, type 2 diabetes, neurodegeneration, autoimmune diseases, and osteoporosis [4,6]. Another undisputed association exists between chronic inflammation and the initiation and progression of cancer [7].

### 1.2. Inflammation and Cancer

Tumor-promoting inflammation has been proposed as an emerging hallmark of cancer by Hanahan and Weinberg in 2011 [8]. First observations linking inflammation to cancer go back to Rudolf Virchow in the 19th century, who observed the presence of tumors noticeably frequent at sites of chronic inflammation and leukocyte infiltration within these cancerous tissues [9,10]. Another hint was the identification of similar inflammatory and tissue remodeling mechanisms that are shared between tumor stroma formation and wound healing processes, which have led to the concept that tumors are “wounds that do not heal” [11,12]. It is now well-established that tumor-infiltrating immune cells can predict survival and therapeutic outcomes in several malignancies such as osteosarcoma, colorectal, or breast cancer (BrCa) [13,14,15,16]. An additional level of complexity is added by the presence of other resident cells and structures within the tumor microenvironment (TME), which are not silent bystanders but are rather fostered (or at least affected) by inflammatory mechanisms, including tumor-associated macrophages (TAM) [17], cancer-associated fibroblasts (CAFs) [18], mesenchymal stromal cells [19], adipocytes [20], and endothelial cells [21]. The diverse cellular profile of the TME gives rise to a highly diverse spectrum of soluble local mediators such as growth factors, cytokines, chemokines, and microRNAs [12,22,23]. The substantially increasing relevance of these observations has fueled the perception that tumors are heterogenous and dynamic formations that need to be considered in their entirety rather than keeping the focus exclusively on the malignant cells [24].

In further consideration, inflammation is the connective link between cancer and its major risk factors including smoking, obesity, hyperglycemia, alcohol consumption, UV light, radiation, as well as chronic bacterial, viral, or parasitic infections, as shown in several studies [25]. All of these factors eventually provoke activation of local or systemic proinflammatory pathways, leading to genomic instability in host cells and consequently predisposing individuals to malignant transformation and directly or indirectly driving malignant cell proliferation and growth [26,27,28,29,30]. The relevance of inflammation in cancer is further underpinned by numerous studies, demonstrating a reduced overall risk of developing cancer by anti-inflammatory agents such as aspirin [31]. One of the most prominent and best-studied transcription factors linking inflammation to cancer is nuclear factor “kappa-light-chain-enhancer” of activated B-cells (NF-κB). This protein is activated by many carcinogens, regulates numerous target genes that are implicated in tumorigenesis, and is constitutively active in most human cancers and pro-tumorigenic inflammatory cells [25]. Moreover, NF-κB is linked to radio- and chemo-resistance and controls a multitude of genes that foster key steps of tumor initiation and progression [25]. Inactivation of NF-κB signaling prevents inflammation-induced tumor formation and metastasis [32]. Two of the most extensively investigated target genes of NF-κB are tumor necrosis factor (TNF)-*α* and interleukin (IL)-6, with both of them playing significant roles in malignancies [33,34,35].

Once a tumor is growing, cancer cells may undergo necrosis as a result of limited nutrient and oxygen supply. Necrotic cells release several molecules that serve as alarming danger-associated molecular patterns (DAMPs), which are not always anti-tumorigenic and protective but may foster inflammation or alter local immune responses by recruitment and modulation of immunosuppressive cells [36,37,38,39]. Moreover, tumors can suppress apoptosis and the cytotoxic host’s immune response [38].

Another hallmark of cancer is metastasis [40], which may occur early in tumor progression [41] and is associated with an increased risk of tumor recurrence and mortality, thus representing one of the main drivers of cancer-related mortality [42]. A prerequisite that allows for leaving the primary tumor is epithelial-to-mesenchymal transition (EMT). EMT is a complex reprogramming network induced and mediated by several triggers and transcription factors and allows epithelial cancer cells to acquire a mesenchymal phenotype. This genetic, metabolic, and morphologic reprogramming of the tumor cells substantially affects several traits that are associated with invasion and dissemination, altered interactions with the extracellular matrix (ECM) and the immune system, and resistance to therapies [30,42,43]. Inflammation and EMT are mutually interacting and sustain each other by creating a positive feedback loop [44]. The most prominent inflammatory mediators that favor EMT and consequently drive the migratory, invasive, and metastasizing potential of tumor cells include IL-1*β*, IL-6, IL-8, TNF-*α*, and a number of chemokines such as CC-chemokine ligand (CCL)2, CCL5, and CCL18 [44]. Local tumor outgrowth at the metastatic site is finally supported by specific education and modification of the TME to create optimal conditions for the survival and engraftment of tumor cells, even before their arrival. This priming of the premetastatic niche, which comprises cell recruitment and restructuring of the ECM, is at least partially mediated by pro-inflammatory cytokines. These are released by the primary tumor or are indirectly activated in the target organ by other tumor-derived soluble factors [45]. Finally, once metastases have been established, inflammatory cytokine and chemokine signaling sustains a tumor-promoting local milieu and fosters further recruitment of pro-tumorigenic cells [46].

In summary, inflammatory processes are a hub in cancer, being involved in virtually all steps of tumorigenesis, from initiation and promotion of primary tumor growth, to EMT, invasion, dissemination, and establishment of solid metastases [24]. In addition, inflammatory cytokines promote angiogenesis [47] and are associated with chemoresistance [48]. Unsurprisingly, an emerging number of preclinical and clinical studies are aimed at identifying the potential of anti-inflammatory agents targeting cytokines and chemokines, transcription factors, or inflammatory cells in patients with cancer (reviewed in [49]).

## 2. Impact of Inflammation on Cancer Metastases to Bone

### 2.1. Bone Metastases

A prime example of metastasis is the dissemination and local outgrowth of tumor cells to and within the bone [50]. The sequential steps of metastasis to bone require physiological reprogramming of the tumor cells and the target TME. The whole process consists of leaving the primary tumor, extravasation, survival in the circulation, and colonization of bone marrow niches, where the cancer cells, now referred to as disseminated tumor cells (DTCs), first need to ensure their survival [51]. In this period of dormancy, tumor cells evade the immune system, adapt to their new microenvironment, and remain in a quiescent state until not yet fully identified re-activating signals induce their outgrowth to clinically manifest metastases [51,52].

The clinical manifestation of bone metastasis is a result of an imbalance between bone formation and bone resorption accompanied by ECM remodeling, leading either to direct destruction of bone tissue (osteolytic metastases) or the production of new bone of poor quality (osteoblastic metastases), both of which contribute to a significantly increased fracture risk in affected individuals [53]. Bone is a mineralized, highly dynamic organ which undergoes continuous remodeling by the concerted action of mainly three cell types: bone-forming osteoblasts, bone-resorbing osteoclasts, and osteocytes. The latter constitute the main cell type within the bone and serve both as mechanosensors and regulators of the bone remodeling process [54]. Bone remodeling is further controlled by direct cell-to-cell contacts and systemic (endocrine) as well as local (autocrine/paracrine) factors [55]. Within bone, the RANKL-RANK-OPG axis is one of the most central regulatory systems in guiding the coupling of bone formation and resorption [56]. Receptor activator of NF-κB ligand (RANKL), produced by and expressed on the surface of osteoblasts, is a potent inducer of osteoclastic differentiation and activity by interacting with its receptor RANK which is expressed on osteoclasts. Osteoprotegerin (OPG), also primarily released by osteoblasts, is a decoy receptor for RANKL, thereby counterbalancing its stimulatory effect on osteoclasts [56,57].

A fine-tuned balance of bone formation and resorption maintains the normal physiology of healthy bone tissue, resulting in a stable bone mass [56]. In the presence of tumor cells, this balance is disturbed, yielding an increased differentiation and activation of osteoblasts and/or osteoclasts. Depending on which of the two processes is favored, tumor cells induce bone metastases that are primarily osteolytic, osteoblastic, or mixed [53,57]. A simplified explanation is provided by the concept of a vicious cycle between bone and tumor cells. Tumor cells secrete factors that directly or indirectly promote bone resorption and suppress osteoblastic bone formation. In turn, growth factors stored within the bone matrix are increasingly released during osteolysis and promote ongoing tumor growth [50,58,59]. One underlying molecular mechanism is the upregulation of RANKL with a concomitant downregulation of OPG. Osteolytic lesions are mainly found in patients with BrCa, whereas prostate cancer (PrCa) bone metastases emerge with the mixed phenotype [60]. Here, tumor cells are able to resemble the phenotype of bone resident cells by the expression of specific transcription factors and the production of bone matrix proteins, an adaptive mechanism referred to as “osteomimicry” [61,62]. Moreover, several secreted proteins of tumor cell-activated osteoblasts increase proliferation, survival, and invasion of the tumor cells [53,59].

Bone metastases impose a significant individual and socio-economic burden and manifest in skeletal-related events (SRE) such as fractures, hypercalcemia, nerve compression syndromes, pain, and immobility [59,63]. Current therapeutic options are limited to pain palliation and the prevention of SRE by surgery, radiotherapy, and pharmaceutical suppression of the high bone turnover by bisphosphonates and the monoclonal RANKL-antibody denosumab [60,64]. While osteolytic lesions secondary to breast cancer are recommended to be treated with antiresorptive agents when diagnosed, antiresorptive treatment in sclerotic lesions may be delayed to later stages when tumors turn refractory to hormone treatment. Additional bone-targeting agents are currently tested in preclinical and clinical studies (reviewed in [63]).

### 2.2. Inflammatory Cellular and Soluble Mediators in Cancer Metastases to Bone Metastases

**Figure 1:**
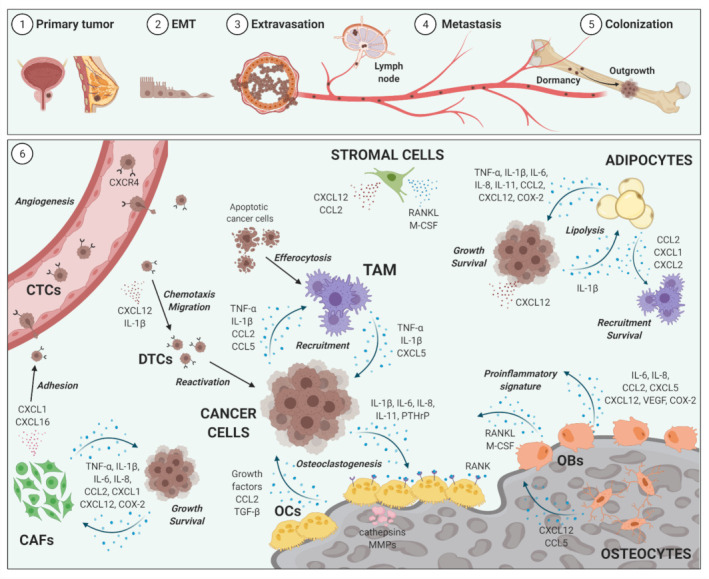
Selected soluble and cellular inflammatory mediators of bone metastasis. Breast and prostate cancer are among the malignancies with the highest propensity to metastasize to bone (**1**). Cancer cells from the primary tumor undergo epithelial-to-mesenchymal transition (EMT) (**2**) and start to extravasate (**3**). Directly detached from the primary tumor or after metastasis to lymph nodes, circulating tumor cells (CTCs) metastasize to bone via the blood stream (**4**) which leads to seeding and colonization of single CTCs within the bone marrow (**5**). After a period of dormancy, disseminated tumor cells (DTCs) start to grow to full-blown metastases (**5**). Several cellular and soluble inflammatory factors maintain and drive the vicious cycle of bone metastases within the tumor microenvironment (**6**). The involved mechanisms are (i) induction of angiogenesis; (ii) mediation of tumor and immune cell recruitment and activation; (iii) maintenance of tumor cell growth and survival; (iv) tumor-promoting education of resident adipocytes, stromal cells, cancer-associated fibroblasts (CAFs), and tumor-associated macrophages (TAM); as well as the acceleration of osteoclastogenesis by inhibiting osteoblastogenesis and driving a local proinflammatory signature. CC-chemokine ligand 2 (CCL2); CC-chemokine ligand 5 (CCL5); C-X-C motif chemokine 1 (CXCL1); C-X-C motif chemokine 2 (CXCL2); C-X-C motif chemokine 12 (CXCL12); C-X-C Motif Chemokine Receptor 4 (CXCR4); cyclooxygenase-2 (COX-2); interleukin-1*β* (IL-1*β*); interleukin-6 (IL-6); interleukin-8 (IL-8); interleukin-11 (IL-11); macrophage colony-stimulating factor (M-CSF); matrix metalloproteinases (MMPs); osteoblasts (OBs); osteoclasts (OCs); osteoprotegerin (OPG); parathyroid hormone-related protein (PTHrP); receptor activator of NF-κB ligand (RANKL); transforming growth factor beta (TGF-β); tumor necrosis factor alpha (TNF-α); vascular endothelial growth factor (VEGF). Created with BioRender.com (accessed date 7 May 2021).

Historical observations of bone involvement in patients with breast cancer by the British surgeon Stephen Paget led him to propose the “seed and soil” theory [65] with the assumption that tumor cells may spread to any organ but can only survive and grow out to full-blown metastases when they land on a suitable microenvironment [65]. The highly vascularized bone marrow microenvironment is an ideal fertile soil for tumor cells to survive and to grow, but also for proinflammatory cells to differentiate, proliferate, and interact with each other in an intensive dialogue [30]. In addition, the cellular composition within bone is quite diverse, with cells arising from hematopoietic (HSCs) and mesenchymal (MSCs) stem cells [66]. Bone serves as a storage for several growth factors such as transforming growth factor (TGF)-*β* and insulin-like growth factors, thus alluring cancer cells to settle and grow [50,67,68]. In addition, released calcium from resorbed bone promotes tumor cell migration and bone metastasis via calcium-sensing receptors [69]. Notably, inflammation contributes to the initiation and progression of bone metastases. Early stages of the metastatic cascade are accompanied by systemic inflammation and a local immunosuppressive milieu within the metastatic site [70]. In addition, bone metastasis is increased in arthritic mice, which also exhibit an induction of proinflammatory cytokines within the bone microenvironment compared to non-arthritic mice [71]. To delineate the connection between inflammation and tumor cell growth within bone, we need to illuminate the heterogeneous composition of cellular and soluble mediators within this specialized niche. We will therefore discuss the role of selected factors, including central components of the vicious cycle (osteoblasts, osteoclasts, tumor cells) and classical inflammatory cytokines (IL-1*β*, TNF-*α*, etc.), as well as cellular and soluble mediators that have attained increasing attention in recent years. (Figure 1.) Certainly, there are several additional players involved that will not be discussed here but should not be neglected and underestimated, such as specific soluble (sex hormones, vitamin D, growth factors, bone matrix and morphogenic proteins, TGF-*β*, Wnt signaling) and cellular mediators (the nervous system, vasculature, HSCs, and cells of the adaptive immune system), etc. [59,60,72,73,74,75].

### 2.3. Selected Cellular Players in Inflammation and Bone-Metastatic Cancer

#### 2.3.1. Osteoblasts and Osteoclasts

Osteolysis is a hallmark of bone metastases secondary to BrCa and characterized by a massive overactivation of osteoclastic bone resorption [62]. Osteoclasts are bone-resorbing cells, whose differentiation depends on several other growth factors, hormones, and cytokines [61]. In the context of bone metastases, several proinflammatory cytokines (IL-1, IL-6, IL-11, TNF-*α*), chemokines (CCL2, CCL3, CCL15, CXCL8/IL-8), and additional factors such as parathyroid hormone-related protein (PTHrP) produced by stromal, immune, or tumor cells directly or indirectly stimulate osteoclastogenesis, thus accelerating the vicious cycle [61,66,76,77]. This can be mediated by stimulating RANKL production in osteoblasts and RANK expression of osteoclast precursors or by RANKL-independent mechanisms [30,78]. RANKL supports bone-homing cancer cell migration, as well as metastasis to and growth within bone [79]. In line, RANK is expressed on primary BrCa cells and cell lines. Neutralization of RANKL/RANK signaling by OPG prevents intraosseous tumor burden in mouse models [80].

Osteoblastogenesis is not only inhibited by tumor-derived factors due to inactivation of pivotal signaling pathways or by induction of osteoblastic and osteocytic apoptosis as shown for Dickkopf-1, IL-6 and TNF-*α* [81,82,83]. In fact, osteoblasts are also educated by cancer cells to favor recruitment and survival of tumor and immune cells as well as to foster angiogenesis. Metastatic cancer cells induce a proinflammatory response in osteoblasts that includes the release of IL-6, IL-8, monocyte chemoattractant protein-1 (MCP-1/CCL2), vascular endothelial growth factor (VEGF), and CXCL5, which further fuel osteoclastic differentiation as well as activity and drive cancer progression [84,85,86,87]. Osteoblasts as a cellular source for C-X-C motif chemokine 12 (CXCL12, also known as stromal cell-derived factor 1, SDF1) are also important for tumor cell homing to bone. This is supported by the finding that tumor cells are often found to selectively disseminate in osteoblast-rich areas of the bone [88]. Increasing attention has recently been devoted to the role of osteocytes in bone metastases. These cells are able to produce inflammatory and chemoattracting mediators such as CXCL12 and IL-11, both of which contribute to enhanced osteoclastogenesis and tumor cell homing to bone [89]. Taken together, inflammation alters the balance between bone formation and resorption by favoring osteoclastogenesis over osteoblastogenesis.

#### 2.3.2. Tumor-Associated Macrophages

Macrophages are central components of the innate immune system with distinct roles in inflammation and tissue homeostasis. Bone marrow resident macrophages contribute to HSC niche maintenance and bone repair [17]. The tumor-promoting roles of macrophages in cancer have been increasingly appreciated, especially as they constitute the major cell type within solid tumors [90]. TAM differ from classically-activated M1 and alternatively-activated M2 macrophages with respect to polarizing signals, cytokine production, and membrane receptors [90,91]. TAM are implicated in tumor growth, invasion, and metastasis as well as angiogenesis and immune evasion mechanisms, and often promote tumorigenesis in a liaison with inflammatory mediators [30,90,91]. They are also involved in early and late stages of bone-metastatic cancer [17,30,91]. This may be illustrated by significantly reduced PrCa and lung cancer cell growth within the bone, a reduction of intratumoral macrophages and osteoclasts in the bone marrow, and an inhibition of osteolytic lesions secondary to systemic depletion of monocytes and macrophages in recipient mice [92,93]. In fact, TAM and osteoclasts are recruited by tumor-derived inflammatory cytokines and chemokines (TNF-*α*, CCL2, CCL5) upon EMT activation and during metastatic outgrowth [30,94,95,96]. The presence of macrophages and tumor cells strongly influences the local signature of soluble factors. Release of proinflammatory CXCL10 and CCL2 by macrophages is stimulated by the contact with tumor cells, which accelerates cancer cell recruitment to and growth within bone as well as osteoclastogenesis [97,98]. Macrophage-derived TNF-*α* and IL-1*β* are strong inducers of IL-6 in tumor cells, a cytokine that in turn promotes intraosseous tumor growth, osteoclastogenesis, and osteolysis [99].

A further key function of macrophages is the engulfment and digestion of apoptotic cells, a process referred to as efferocytosis, that normally leads to production of anti-inflammatory cytokines. However, efferocytosis of tumor cells initiates NF-κB and signal transducer and activator of transcription 3 (STAT3) signaling, thus activating proinflammatory cytokine production, especially CXCL5. This chemokine, in turn, accelerates efferocytosis and PrCa development [100,101]. CXCL5 serum levels are significantly increased in patients with PrCa and bone metastases compared to healthy controls or patients with localized PrCa only [102]. Induction of apoptosis in M2-like macrophages by Trabectedin significantly reduces skeletal metastasis of PrCa cells [101]. These observations prove that tumor cell death can still promote tumor cell survival and growth, at least by education of the inflammatory TAM machinery. Encouraging recent studies have demonstrated that the administration of anti-inflammatory and pro-resolving lipid mediators are able to prevent this mechanism, thereby suppressing tumor growth and improving chemotherapy [103].

#### 2.3.3. Bone Marrow Adipocytes

Obesity is tightly linked to chronic inflammation [104] and associated with risk for developing tumors and metastatic spread in several malignancies including BrCa and PrCa [105,106,107,108,109]. Bone marrow adipocytes are metabolically active cells which store and secrete a diverse panel of cytokines, fatty acids, and hormones (adipokines) and are able to affect bone and other neighboring cells by paracrine and endocrine mechanisms [108,110]. These cells are among the most abundant cell types present in the bone marrow [111]. Upon obesity, the number of bone marrow adipocytes increases [112]. Adipokines mediate several protumorigenic effects on cancer cells, including promotion of survival, growth, invasion, migration, and metastasis, as well as the recruitment of immune cells and mediation of chemoresistance [110,113,114].

Adipocytes and osteoblasts within the bone marrow share common stromal progenitor cells. While the bone marrow adipocyte number increases with age, the number of osteoblasts progressively declines, implying an imbalance in differentiation between the two lineages with growing age [108]. Moreover, adipocyte-secreted factors activate an inflammatory pro-osteoclastic signature within the bone while pathways fueling osteoblastogenesis are inhibited [108,115,116,117,118]. Bone marrow adipocytes and adipokines attract invading tumor cells, are often found in close proximity to them, and further stimulate tumor cells to activate NF-κB signaling and to produce inflammatory cytokines and chemokines [119,120,121]. Unsurprisingly, bone marrow adiposity is linked to reduced bone volume and increased bone-degrading proteases and inflammatory cytokines, as well as skeletal metastasis [108,118,122,123]. Moreover, obesity is associated with the increased production of CCL2 and cyclooxygenase-2 (COX-2) within the bone marrow, factors which, in turn, exert protumorigenic and proosteoclastic effects [108]. The production of COX-2 by bone marrow adipocytes promotes osteolysis and is involved in evasion mechanism of tumor cells by creating an immune suppressive milieu [113]. PrCa and BrCa cells incorporate adipocyte-derived factors such as fatty acids which support tumor growth and invasion. This is at least partly mediated by stimulation of IL-1*β* expression, which consequently fuels PrCa cell engraftment within the bone marrow [120,122,124,125,126]. A coculture of adipocytes with BrCa cells both stimulates tumor cell invasion and modulates a pro-inflammatory reprogramming of adipocytes [127]. Moreover, adipocyte-derived chemotactic CXCL1 and CXCL2, ligands of the CXCR2 receptor, stimulate macrophage recruitment and osteoclastogenesis, thus sustaining the intraosseous vicious cycle [108,123]. Hence, several inhibitors of adipokines are currently investigated in clinical and preclinical trials for their anti-tumor effects as well as their potential to suppress bone metastasis [113].

#### 2.3.4. Cancer-Associated Fibroblasts

CAFs are heterogeneous tumor-promoting cells of the stromal part and emerging players within solid tumors and implicated in EMT, modulation of ECM, angiogenesis, tumor progression and survival, and metabolic reprogramming of tumor cells, as well as chemotherapy resistance [18,128,129]. Apart from normal resident fibroblasts, a number of additional cell types are able to differentiate into CAFs, such as epithelial cells, pericytes, and smooth muscle cells, as well as bone marrow-derived and/or tumor-associated MSCs [130]. Metastasis site-specific functions and phenotypes of CAFs have been suggested as heterogeneous histological patterns of CAFs-related proteins have been found in brain, liver, lung, and bone metastases secondary to BrCa [131]. CAFs found in metastases of skin, ovarian, and pancreatic cancer, as well as BrCa, express a pro-inflammatory gene signature, thereby promoting tumor cell survival and recruitment of protumorigenic immune cells. Consistently, NF-κB reflects a master regulator of this signature [129]. The generation and activation of CAFs within tumors is at least partially mediated by inflammatory mediators and transcription factors (IL-1, IL-6, NF-κB) as part of an intensive dialogue with tumor cells [18,128,132]. Evidence also exists for a role of CAFs in bone metastasis [133]. CAFs in the primary tumor are able to express high levels of CXCL12, CXCL1, and insulin-like growth factor 1 (IGF1). These factors favor the selection of tumor cell clones which are primed to metastasize to and adhere to bone, a microenvironment being itself rich in CXCL12 [134,135]. CXCL1 and CXCL16 released by PrCa-associated fibroblasts stimulate tumor cell adhesion within the bone matrix [135]. In addition, CAFs are able to sense DAMPs, which ultimately activate the inflammasome to promote IL-1*β* production, which fuels BrCa tumor growth and metastasis [136]. Hence, inflammatory mechanisms in the primary tumor are able to affect the metastatic fate of released tumor cells. Moreover, experimental models with CAFs isolated from bone metastases illuminated a role of oncogenic miR-221-containing CAFs microvesicles in inducing hormonal therapy resistance in human BrCa cells, a mechanism that was mainly sustained by autocrine IL-6 signaling [137]. CAFs-derived IL-6 was further shown to mediate resistance to the cytotoxic effects of doxorubicin in PrCa cells [138]. These observations may identify CAFs as a promising target in bone-homing malignancies.

#### 2.3.5. Selected Inflammatory Cytokines, Chemokines and Mediators

As previously described, different cell types are involved in cancer metastasis to bone. All of them are able to produce a broad panel of soluble mediators [139,140], some of which are summarized in Table 1.

#### 2.3.6. TNF-*α*

TNF-*α* is a classical pro-inflammatory cytokine produced by activated monocytes, macrophages, CAFs, adipocytes, and tumor cells, among others [141]. TNF-*α* mediates both pro- and anti-tumorigenic effects in a context-dependent fashion. However, a tumor-supporting role is believed to predominate as TNF-*α* has been shown to support tumor progression and metastasis, angiogenesis, and resistance to immunotherapies [9,34,110,142]. TNF-*α* is implicated in primary stages of bone-metastatic malignancies including BrCa and PrCa, where it is often overexpressed and supports maintenance of stem-like cells [143]. Increased levels of TNF-α are found in patients with metastasized PrCa [144]. Strong interactions exist between TNF-*α* and further mediators such as CCL2, whose expression by tumor cells and macrophages is directly stimulated by TNF-α, thus sustaining a tumor-promoting effect [145]. Moreover, TNF-*α*-activated mesenchymal stromal cells activate a proinflammatory chemokine signature that recruits neutrophils, which in turn stimulate the initiation of a metastatic reprogramming of tumor cells [146]. TNF-*α* leads to reduced OPG production, thereby stimulating osteoclastogenesis and tumor growth. Neutralization of TNF-α suppresses osteolysis by the BrCa cell line MDA-MB-231, an effect that involves a reduction of CXCL12/CXCR4 signaling [78,147].

#### 2.3.7. IL-1*β* and NF-κB Signaling

The pro-inflammatory cytokine IL-1*β* is a key mediator within the inflammatory TME, where it is produced by several cell types including fibroblasts, adipocytes, TAM, and tumor cells [113,148]. IL-1*β* is a member of the IL-1 family, secreted as an inactive pro-protein and cleaved by inflammasome-activated caspase-1 into a mature, biologically active form [148,149]. IL-1*β*/IL-1R signaling induces a wide range of effects which are mediated by activation of signaling proteins such as NF-κB [148]. IL-1*β* has been shown to be significantly upregulated in most primary tumors such as breast, prostate, colon, lung, head and neck cancer, and melanoma and promotes tumor cell migration, angiogenesis, immune response, and metastasis formation [148,150]. High levels of plasma IL-1*β* and tumor-derived IL-1*β* significantly correlate with tumor invasiveness and bone metastasis in BrCa and PrCa, which translates into a poor prognosis [151,152,153,154,155]. Consequently, tumor cell-derived IL-1*β* has been identified as a potential biomarker for predicting the risk of developing bone metastases in patients with BrCa and is an emerging therapeutic target in bone-homing malignancies [156]. IL-1*β* promotes cancer metastasis to bone by increasing EMT and tumor cell invasion and migration to the bone marrow and adipose tissue [120,151,154,157,158]. In models of dormancy, IL-1*β* together with TNF-*α* stimulated the reactivation and proliferation of tumor cells within an osteoblastic matrix [159]. Moreover, IL-1*β* induces lipolysis of adjacent adipocytes and the expression of metastasis-related and inflammatory factors such as matrix metalloproteinases (MMP), COX-2, CCL2, IL-6, TGF-*β*, and IL-8 which promote tumor growth and bone metastasis [140,151,160,161]. Targeting IL-1*β* signaling, either genetically or pharmacologically using the neutralizing IL-1*β* antibody canakinumab or the IL1R antagonist Anakinra, significantly reduced experimental BrCa and PrCa bone metastases [154,157,162,163]. These effects were accompanied by diminished activation of osteoclasts and reduced TNF-*α* release, further indicating the potential of targeting IL-1*β* in breaking the vicious dialogue between tumor and resident bone cells [157].

A direct link between bone marrow-derived IL-1*β* and one of its main downstream transcription factors, NF-κB, has been demonstrated to favor BrCa stem cell colonization and outgrowth within bone [162]. Indeed, activated NF-κB signaling as well as interacting signaling axes such as STAT3 and downstream mediators are considered as one of the main culprits in tumorigenesis [164]. A strong NF-κB/STAT3 interaction with downstream inflammatory targets ensures continuous positive feedback loops resulting in signal amplifications within tumors [30]. Concerning BrCa and PrCa cells, NF-κB and STAT3 are associated with premetastatic niche formation and migration and metastasis to the bone, as well as growth within the bone, at least partially by stimulating RANKL and PTHrP production, thereby accelerating tumor cell-promoted osteoclastogenesis [165,166,167,168,169,170]. Notably, NF-κB increases the expression of vascular cell adhesion molecule (VCAM)1 in bone micrometastasis. This protein, together with *α*4*β*1 integrin upregulation, recruits osteoclast precursors, accelerates local osteoclastogenesis, and drives the progression into full-blown osteolytic macrometastases [30,171]. Constitutive activation of NF-κB in PrCa involves the activity of miR-210-3p, which is found to be significantly upregulated in bone-metastatic PrCa [167]. These observations again highlight the strong local interactions of several soluble mediators that create a pool of tumor-promoting signals.

#### 2.3.8. IL-6, CXCL-8/IL-8, IL-11

Several tumor cells including BrCa and PrCa cells as well as local cells of the TME such as adipocytes, macrophages, or osteoblasts are able to produce IL-6, IL-8, and IL-11. In turn, tumor cells also induce inflammatory cytokine production in osteoblasts [84,86,87,172]. In primary and metastatic BrCa and PrCa, increased levels of these cytokines correlate with the extent of the disease and are associated with poor survival and chemotherapy resistance [144,173,174,175,176]. IL-6 and IL-8 have been shown to promote a process referred to as “tumor self-seeding”, where CTCs are recruited back to the primary tumor where they modify progression and metastatic fate of cancer cells [177]. Moreover, they are found to be elevated in the serum of patients with bone metastases secondary to BrCa and PrCa and directly or indirectly activate osteoclastogenesis, RANKL and PTHrP expression, angiogenesis, and tumor growth, thereby fueling the osteolytic vicious cycle [99,110,163,172,176,178,179,180,181,182,183,184,185,186,187]. Cancer cell-, osteoblast-, or adipocyte-derived IL-6 and IL-8 directly or indirectly promote EMT, angiogenesis, tumor cell growth, migration, and metastasis, as well as CAFs activation by interacting with additional inflammatory pathways [132,188,189,190,191,192]. Moreover, IL-6 upregulates the expression of the androgen receptor and of CXCR4 on tumor cells, thereby favoring tumor cell proliferation and recruitment to bone via the CXCL12/CXCR4 axis [193,194]. Among other factors, increased CXCR4 and IL-11 expression has been identified as a characteristic bone metastatic gene signature [195]. Genetic or pharmacological inhibition of IL-6, IL-8, and IL-11 or downstream mediators in models of bone metastases significantly decreases tumor growth and survival and osteolysis and prolongs survival of tumor-bearing mice [121,163,181,185,196,197,198], highlighting the importance of these mediators in cancer metastasis to bone.

#### 2.3.9. CXCL12/SDF-1

The role of chemokines and chemokine receptors has gained increasing attention over the past 20 years, and CXCL12 is one of the most studied [78]. Mediating its effects by binding to its major corresponding receptor, CXCR4, CXCL12 is implicated in inflammatory responses, autoimmune diseases, T cell homing, angiogenesis, CAFs activation, EMT, tumor progression, metastasis, and therapy resistance [199,200,201,202]. A high expression and activation of the CXCL12/CXCR4 axis can be found at preferred metastatic sites such as lymph nodes, liver, lungs, and the bone marrow associated with poor prognosis [195,203,204,205]. In bone metastases, CXCL12 and CXCR4 can be produced or expressed by a broad panel of cell types such as osteoblasts, osteocytes, CAFs, neutrophils, tumor cells, and adipocytes [47,113,206,207,208,209]. A high expression of CXCR4 in BrCa and PrCa tissue or tumor cells significantly correlates with distant lymph node, lung, and bone metastases, and increased aggressiveness [210,211,212]. Here, similarly to HSC recruitment, CXCL12/CXCR4 signaling is one of the main stimulatory axes attracting CTCs to home to and to adhere within bone, and also supports tumor cell survival and dormancy as well as angiogenesis [19,47,52,213]. It has been shown that tumor-derived angiopoietin-like protein 2 increases CXCR4 expression on tumor cells, thus increasing their responsiveness to CXCL12 [214]. The importance of CXCL12/CXCR4 in intraosseous tumor cell dissemination is further underpinned by the observation that inhibition of CXCR4 remobilizes PrCa DTCs back into circulation, thus possibly increasing their vulnerability to anticancer treatment [215,216]. Experiments in zebrafish have further delineated a role of host neutrophil CXCR4 signaling in early progression of BrCa micrometastases [209]. CXCL12 is in direct crosstalk with additional inflammatory cytokines such as IL-17A. In arthritic mice, bone metastasis could be prevented by neutralizing IL-17A, and local reduction of bone-derived CXCL12 was proposed as one of the underlying mechanisms [217]. In multiple myeloma, tumor cell-derived CXCL12 stimulates bone resorption, providing a potential explanation for the positive correlation between CXCL12 plasma levels and osteolytic lesions in affected patients [218]. Indeed, genetic CXCL12 deletion or pharmacological inhibition of CXCR4 partially prevents BrCa and PrCa metastasis to lungs and bones as well as early steps of intraosseous tumor growth [203,204,219,220,221].

#### 2.3.10. CCL2/MCP-1

CCL2 or MCP-1 is an inflammatory cytokine produced by osteoblasts, osteoclasts, tumor, endothelial, and stromal cells, and is further stimulated within the TMA by bone marrow adiposity, inflammation, and the presence of tumor cells or tumor-derived factors such as PTHrP [108,222,223,224,225]. CCL2 is not only one of the most upregulated genes in osteoporotic bone, but is also implicated in the regulation of skeletal metastasis by increasing osteoclastic differentiation [96,108,226,227]. Moreover, it exerts pleiotropic protumorigenic effects, including direct promotion of tumor cell growth, migration, and metastasis, but also indirectly by priming a tumor-supportive metastatic niche via angiogenesis, TAM recruitment, and triggering of a proinflammatory milieu [223,228,229,230]. In concert with IL-6, CCL2 increases the survival of macrophages and stimulates their differentiation towards a tumor-promoting M2-like phenotype [231]. CCL2 is significantly upregulated in primary BrCa, where it is associated with an proinflammatory signature, TAM recruitment, NF-κB activation, poor prognosis, and the risk of early relapse [232,233,234]. In PrCa, CCL2/CCR2 expression correlates with bone metastases in patients and in preclinical models [96,223,235,236]. Cross-signaling between BrCa and stromal cells via CCL2/CCR2 stimulates the recruitment of inflammatory monocytes and osteoclastic differentiation, thus favoring pulmonary and bone metastasis of BrCa cells, respectively [84,86,95,96,237]. Neutralization of CCL2 prevents tumor cell migration, osteolysis, and angiogenesis in mouse models of bone-metastatic PrCa and BrCa [95,222,238,239]. Notably, CCL2 is involved in the progression of skeletal metastasis elicited by a local inflammatory activation and the expansion of myeloid cells in the bone marrow by cyclophosphamide [240]. Based on these investigations, clinical studies using CCL2 or CCR2 antibodies were conducted. However, mostly discouraging results indicate that the role of CCL2/CCR2 signaling in bone metastases is likely to be more complex than initially anticipated. Indeed, some studies have proposed an inhibitory role of CCL2 in the bone-metastatic process, thus highlighting the importance of further investigation to tailor targeted therapies [241,242].

#### 2.3.11. CCL5/RANTES

Similar to CCL2, CCL5 is weakly expressed in healthy breast tissue, but its expression increases in BrCa, especially in advanced stages, where it is also significantly enriched in the plasma of affected patients [243,244]. By favoring TAM recruitment and by cooperating with CCL2, CCL5 drives BrCa recurrence [245] and bone metastatic progression [229]. Here, CCL5 also supports osteoclastogenesis [226]. Interestingly, BrCa cells stimulate CCL5 production by MSCs, which in turn stimulates tumor growth, invasion, and metastasis, a process that may occur in bone metastasis, too [246]. CCL5 and MMP expression is increased in mechanosensing osteocytes as a consequence of increased tumor burden and intraosseous pressure, thus facilitating PrCa growth and invasion [247]. In addition, TAM-derived CCL5 supports invasion, growth, and self-renewal of PrCa stem cells. Knockdown of CCL5 in TAM reduces prostate cancer growth and bone metastasis in vivo [248].

#### 2.3.12. Cyclooxygenase-2 and Prostaglandins

COX-2 catalyzes the production of prostaglandins from arachidonic acid, is implicated in several steps of tumorigenesis, and is induced in inflamed and cancerous tissues by proinflammatory cytokines including IL-1*β* and TNF-*α* [249,250]. The COX-2 derived prostaglandins induce the production of several pro-osteoclastic mediators including inflammatory cytokines IL-1, IL-6, IL-11, and TNF-*α* [251]. Increased COX-2 expression is linked to cancer progression and metastases and often coincides with an overexpression of CCL2 [59,108,250]. In BrCa, high COX-2 expression positively correlates with the presence of bone metastases and accelerated osteoclastogenesis [252,253,254]. In addition, COX-2 expression in osteoblasts is induced by the contact with BrCa cells and leads to autocrine stimulation of RANKL production [255]. Neutralization of prostaglandins, antagonizing prostaglandin receptors, and the use of COX-2 inhibitors such as Celecoxib significantly reduce the risk of bone metastases, decrease osteolysis and reduce the levels of IL-1*β*, IL-6, and COX-2 within osteolytic lesions, respectively [108,256,257,258,259,260].

**Table 1:** Role of selected inflammatory cellular and soluble mediators in cancer metastasis to bone.

Mediator	Cellular Source	Mechanism of Action in Cancer Metastasis to Bone	Selected References
**CCL2/MCP-1**	Adipocytes, ECs, OBs, OCs, TAM, stromal cells, tumor cells	Angiogenesis↑; COX-2↑; EMT↑; NF-κB activation↑; osteoclastogenesis↑; TAM recruitment and activation↑; tumor cell recruitment, growth, migration, metastasis↑;	[44,86,95,96,108,145,222,223,224,225,226,227,228,229,230,231,232,233,234,235,236,237,238,239,240,241,242]
**CCL5**	Osteocytes, stromal cells, TAM, tumor cells	EMT↑; Osteoclastogenesis↑; TAM recruitment and activation ↑; tumor cell recruitment, growth, migration, metastasis↑	[44,226,229,243,244,245,246,247,248]
**COX-2/** **prostaglandins**	Adipocytes, OBs, TAM, tumor cells	CCL2↑; IL-8↑; IL-11↑; immunosuppression↑; osteoclastogenesis↑; prostaglandin E2↑; TAM recruitment and activation↑; tumor cell growth, migration, metastasis↑	[59,108,113,249,250,251,252,253,254,255,256,257,258,259,260]
**CXCL12/SDF1**	Adipocytes, CAFs, neutrophils, OBs, osteocytes; stromal cells, tumor cells	Angiogenesis↑; CAFs generation↑; chemotherapy resistance↑; dormancy↑; EMT↑; osteoclastogenesis↑; tumor cell recruitment, growth, migration, metastasis↑	[78,88,89,147,193,194,195,199,200,201,202,203,204,205,206,207,208,209,210,211,212,213,214,215,216,217,218,219,220,221]
**IL-1*β*/NF-κB**	Adipocytes, CAFs, OBs, OCs, TAM, tumor cells	Adipocytic lipolysis↑; CAFs generation↑; CCL2↑; COX-2↑; dormancy↑; EMT↑; IL-6↑; IL-8↑; osteoclastogenesis↑; premetastatic niche formation↑; tumor cell recruitment, growth, migration, metastasis↑	[44,81,113,120,140,148,149,150,151,152,153,154,155,156,157,158,159,160,161,162,163,164,165,166,167,168,169,170,171]
**IL-6**	Adipocytes, CAFs, OBs; OCs, stromal cells, TAM, tumor cells	Angiogenesis↑; CAFs generation↑; CXCR4↑; chemotherapy resistance↑; COX-2↑; EMT↑; IL-8↑; IL-11↑; osteoblastogenesis↓; osteoclastogenesis↑; PTHrP↑; RANKL↑; tumor cell recruitment, growth, migration, metastasis↑	[44,77,84,86,87,99,110, 121,132,138,144,163, 172,173,174,175,176,177,178,179,180,181,182,183,184,185,186,187,188,189,190,191,192,193,194,196,197,198]
**IL-8/CXCL8**	Adipocytes, CAFs, ECs, OBs, TAM, tumor cells	Angiogenesis↑; CAFs generation↑; chemotherapy resistance↑; EMT↑; osteoclastogenesis↑; PTHrP↑; RANKL↑; tumor cell recruitment, growth, migration, metastasis↑	[44,84,86,87,99,110,132,144,163,172,173,174,175,176,177,178,179,180,181,182,183,184,185,186,187,188,189,190,191,192,198]
**IL-11**	Adipocytes, OBs, osteocytes; TAM; tumor cells	Chemotherapy resistance↑; osteoblastogenesis↓; OPG↓; osteoclastogenesis↑	[77,84,86,87,89,144,179,180,181,182,183,184,185,186,187,195]
**TNF*α***	Adipocytes, CAFs, ECs, neutrophils, TAM, tumor cells	Angiogenesis↑; chemotherapy resistance↑; dormancy↑; EMT↑; IL-6↑; osteoblastogenesis↓; osteoclastogenesis↑; OPG↓; TAM recruitment and activation↑; tumor cell recruitment, growth, migration, metastasis↑	[9,33,34,35,44,78,99,110,141,142,143,144,145,146,147,159]

Cancer-associated fibroblasts (CAFs); CC-chemokine ligand 2 (CCL2); CC-chemokine ligand 5 (CCL5); chemokine (C-X-C motif) ligand 8 (CXCL8); C-X-C motif chemokine 12 (CXCL12); cyclooxygenase-2 (COX-2); endothelial cells (ECs); epithelial-to-mesenchymal transition (EMT); interleukin-1*β* (IL-1*β*); interleukin-6 (IL-6); interleukin-8 (IL-8); interleukin-11 (IL-11); monocyte chemoattractant protein-1 (MCP-1); nuclear factor “kappa-light-chain-enhancer” of activated B-cells (NF-κB); osteoblasts (OBs); osteoclasts (OCs); osteoprotegerin (OPG); parathyroid hormone-related protein (PTHrP); receptor activator of NF-κB ligand (RANKL); stromal cell-derived factor 1 (SDF1); tumor-associated macrophages (TAM); tumor necrosis factor alpha (TNF-*α*). ↑ upregulation/activation ↓ downregulation/inhibition.

## 3. Outlook

Inflammation and cancer are inseparably linked. Inflammatory mediators drive tumor initiation, progression, and metastases as well as chemoresistance at several levels, making them attractive therapeutic targets. Bone metastases secondary to BrCa and PrCa are a serious, incurable, long-term complication and associated with the risk of fracture, immobility, and pain. Currently, standard therapeutic options are limited to the prevention of SRE and pain palliation by surgery, radiotherapy, and the use of bisphosphonates and the monoclonal RANKL-antibody denosumab. Research needs to further delineate the importance of inflammatory mechanisms during specific stages of bone metastasis and to expand these investigations by characterizing the specific role of additional cellular and soluble players, such as neutrophils or osteocytes, and by revealing unidentified mutual interactions. Moreover, a precise definition of when and how these mediators contribute to bone metastases at different stages and in different cancer subtypes is necessary. This is of special interest as the therapeutic success of targeting inflammatory mediators is likely to be dependent on additional parameters such as host metabolism, environmental factors, cancer phenotype, the specific cellular composition of stromal, immune and tumor cells within the metastatic niche, and the therapeutic window.

## Data Availability

Not applicable.

## Abbreviations

Breast cancer (BrCa); cancer-associated fibroblasts (CAFs); CC-chemokine ligand 2 (CCL2); CC-chemokine ligand 5 (CCL5); chemokine (C-X-C motif) ligand 8 (CXCL8); C-X-C motif chemokine 12 (CXCL12); C-X-C Motif Chemokine Receptor 4 (CXCR4); circulating tumor cells (CTCs); cyclooxygenase-2 (COX-2); danger-associated molecular pattern (DAMP); disseminated tumor cells (DTCs); epithelial-to-mesenchymal transition (EMT); extracellular matrix (ECM); hemopoietic stem cells (HSCs); interleukin-1*β* (IL-1*β*); interleukin-6 (IL-6); interleukin-8 (IL-8); interleukin-11 (IL-11); matrix metalloproteinase (MMP); mesenchymal stem cells (MSCs); monocyte chemoattractant protein-1 (MCP-1); multiple myeloma (MM); nuclear factor “kappa-light-chain-enhancer” of activated B-cells (NF-κB); osteoprotegerin (OPG); parathyroid hormone-related protein (PTHrP); pathogen-associated molecular pattern (PAMP); prostate cancer (PrCa); receptor activator of NF-κB ligand (RANKL); signal transducer and activator of transcription 3 (STAT3); skeletal-related event (SRE); stromal cell-derived factor 1 (SDF1); tumor-associated macrophages (TAM); transforming growth factor beta (TGF-*β*); tumor microenvironment (TME); tumor necrosis factor alpha (TNF-*α*); vascular endothelial growth factor (VEGF).
